# Targeting binding partners of the CBFβ-SMMHC fusion protein for the treatment of inversion 16 acute myeloid leukemia

**DOI:** 10.18632/oncotarget.11357

**Published:** 2016-08-17

**Authors:** Lisa Richter, Yiqian Wang, R. Katherine Hyde

**Affiliations:** ^1^ Department of Biochemistry and Molecular Biology and the Fred and Pamela Buffett Cancer Center, University of Nebraska Medical Center, Omaha, NE, USA

**Keywords:** inv(16), CBFβ-SMMHC, CBFβ, RUNX1, AML

## Abstract

Inversion of chromosome 16 (inv(16)) generates the CBFβ-SMMHC fusion protein and is found in nearly all patients with acute myeloid leukemia subtype M4 with Eosinophilia (M4Eo). Expression of CBFβ-SMMHC is causative for leukemia development, but the molecular mechanisms underlying its activity are unclear. Recently, there have been important advances in defining the role of CBFβ-SMMHC and its binding partners, the transcription factor RUNX1 and the histone deacetylase HDAC8. Importantly, initial trials demonstrate that small molecules targeting these binding partners are effective against CBFβ-SMMHC induced leukemia. This review will discuss recent advances in defining the mechanism of CBFβ-SMMHC activity, as well as efforts to develop new therapies for inv(16) AML.

## INTRODUCTION

Acute myeloid leukemia (AML) is often classified by the presence of specific, recurrent chromosomal abnormalities that generate characteristic fusion genes [1-3]. Expression of these fusion genes is likely the initiating event, although additional cooperating mutations are required for transformation to a frank leukemia. While the profile of cooperating mutations may vary in different leukemia sub-clones, the specific fusion protein involved is likely present in all leukemia cells of a given patient, making them attractive therapeutic targets [1, 4, 5]. However, many leukemia fusion proteins involve transcriptional regulators, which have historically been difficult to target. Recently, multiple inhibitors that target fusion proteins have been described, including 3 different drugs targeting the fusion protein expressed in inversion 16 AML [6-10].

**Figure 1:**
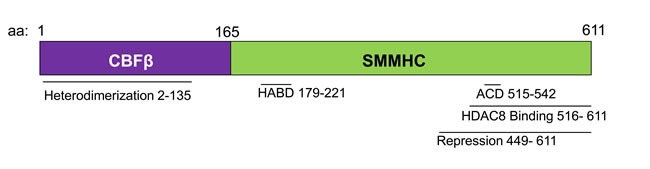
Schematic representation of the CBFβ-SMMHC fusion protein Diagram representing the indicated domains of the fusion protein, and the associated amino acid (aa) numbers. HABD: High Affinity Binding Domain. ACD: Assembly Competence Domain.

Inversion of chromosome 16, inv(16)(p13q22) (inv(16)) and the related, but less common translocation t(16;16)(p13;q22), are associated with nearly all cases of French-American-British (FAB) classified AML subtype M4 with Eosinophilia (M4Eo) [11, 12]. Both of these rearrangements generate a fusion between the gene for Core Binding Factor β (*CBFB*) and the *MYH11* gene, which encodes Smooth Muscle Myosin Heavy Chain (SMMHC) (Figure 1) Expression of *CBFB-MYH11* is thought to be the initiating event in inv(16) AML [13, 14].

Inv(16) and t(16;16) also generate the reciprocal *MYH11-CBFB* fusion gene. However, this region is lost in some M4Eo AML patients, with no discernable clinical effect. Consequently, the *MYH11-CBFB* fusion is thought to be dispensable for leukemia development [15]. This is in contrast to other reciprocal chromosomal rearrangements, such as t15;17, which generates the PML-RARα and RARα-PML fusions, and t(4;11)(q21;q23), which generates the MLL-AF4 and AF4-MLL fusions. Both products of these chromosomal rearrangements are known to contribute to leukemogenesis [16-19].

Studies in mice indicate that expression of *CBFB-MYH11*, which encodes the protein CBFβ-SMMHC, is necessary, but not sufficient for AML development [20, 21]. CBFβ-SMMHC alone causes defects in hematopoietic differentiation, but additional cooperating mutations are required for transformation to frank leukemia [22-28].

Inv(16) is found in 8-10% of pediatric and adult AML cases [2, 29, 30]. Inv(16) is considered a good prognosis AML due to the high rate of response to current treatments [3, 31]. Nearly 90% of inv(16) AML patients achieve clinical remission under current chemotherapy protocols, although approximately half will eventually relapse [32-35]. In addition, current treatments are associated with significant morbidity and mortality [36-38].

## CBFβ-SMMHC AND RUNX1

CBFβ is a member of the Core Binding Factor (CBF) family of transcription factors, and heterodimerizes with the DNA binding α subunits, RUNX1, RUNX2, and RUNX3 [39, 40]. CBFβ does not directly bind DNA, but stabilizes the interaction between the α subunits and DNA, and protects them from degradation [39, 41, 42].

RUNX1 is a key transcription factor during hematopoiesis. During embryogenesis, RUNX1 is required for the generation of hematopoietic stem cells (HSCs) from the hemogenic epithelium and for the proper differentiation of primitive erythrocytes [43-46]. During adult hematopoiesis, RUNX1 regulates HSC self-renewal and survival, as well as the differentiation of megakaryocytes, T-, and B-cells [47-51].

RUNX1 is also associated with a variety of hematological malignancies [52-58]. Point mutations and deletions of RUNX1 are frequently found in patients with myelodysplastic syndrome (MDS), chronic myelomonocytic leukemia (CMML) and FAB M0 AML [59-62]. Heterozygous germline mutations in *RUNX1* cause Familial Platelet disorder with a predisposition to AML (FPD-AML) [63, 64]. Translocations involving RUNX1, t(8;21)(q22;q22) and t(12;21)(p13;q22), are associated with M2 AML and acute lymphoblastic leukemia (ALL), respectively [65-69].

### Dominant negative model of CBFβ-SMMHC activity

CBFβ-SMMHC retains the ability to bind RUNX1 through the N-terminal half of the fusion protein (Figure 1) [42, 70]. In addition, there is a high-affinity binding domain (HABD) in the SMMHC tail. This allows the fusion protein to bind RUNX1 at two sites and outcompete wildtype CBFβ for RUNX1 binding [71]. Because of RUNX1′s established role in hematopoiesis and leukemogenesis, it has been proposed that CBFβ-SMMHC acts as a dominant repressor of RUNX1 [71-73].

Early studies in mice demonstrate that CBFβ-SMMHC dominantly represses RUNX1 in vivo. Knockin mice with a single copy of the fusion gene expressed from the endogenous *Cbfb* promoter (*Cbfb^+/MYH11^*) have a phenotype remarkably similar to mice homozygous for null alleles of Runx1 (*Runx1^−/−^*) or Cbfb (*Cbfb^−/−^*) [43, 44, 74-77]. Embryos of all three of these genotypes have a complete block in definitive hematopoiesis, central nervous system hemorrhaging, and embryonic lethality between embryonic day 12.5 and 13.5 (e12.5- e13.5). These findings indicate that CBFβ-SMMHC represses RUNX1 activity, consistent with a dominant negative model of CBFβ-SMMHC activity [43, 44, 74-77]. However, more recent work has demonstrated that the fusion protein has additional activities, as well.

### CBFβ-SMMHC and RUNX1 independent activities

During primitive hematopoiesis, the initial wave of embryonic blood development that generates primarily nucleated erythrocytes, *Cbfb^+/MYH11^* embryos have differentiation defects that are not seen in either *Runx1^−/−^* or *Cbfb^−/−^* embryos [74, 78]. At e10.5, *Runx1^−/−^* embryos have a subtle differentiation defect, resulting in a small population of circulating immature erythrocytes [46]. Cbfb+/MYH11 embryos, have a significantly more severe differentiation defect, with cells arrested at an earlier stage of differentiation and a larger population of circulating immature cells [78]. This indicates that *Cbfb-MYH11* has RUNX1 repression-independent activities during primitive hematopoiesis.

*Cbfb^+/MYH11^* embryos also have changes in gene expression that are not observed in *Cbfb^−/−^* mice. Microarray analysis of peripheral blood from *Cbfb^+/MYH11^* embryos identified deregulated expression of 658 genes, while the same analysis of *Cbfb^−/−^* embryos identified only 174 differentially expressed genes, with only 71 genes deregulated in both *Cbfb^+/MYH11^* and *Cbfb^−/−^* embryos [78]. The majority of the genes deregulated in both *Cbfb^+/MYH11^* and *Cbfb^−/−^* embryos showed increased expression (95% and 77%, respectively). Importantly, many of the genes that showed deregulated expression uniquely in *Cbfb^+/MYH11^* embryos are also expressed in inv(16) patient samples [78]. This indicates that CBFβ-SMMHC has effects on gene expression that are not due to loss of the RUNX1 activity, and that RUNX1 repression-independent activities may be important for leukemia development.

Clinical data from inv(16) AML patients is also consistent with the CBFβ-SMMHC fusion protein having RUNX1 repression-independent activities. If dominant repression of RUNX1 were CBFβ-SMMHC's only activity, one would expect that loss of *RUNX1* would result in leukemia with similar characteristics to those with inv(16). Instead, *RUNX1* point mutations are associated with stem cell-like, M0 AML with poor prognosis, while expression of CBFβ-SMMHC is associated with a more differentiated, myelomonocytic M4 AML with relatively good prognosis [32-35, 59-61]. These differences in clinical presentation and outcome imply fundamental differences in the underlying leukemogenic process of these two AML subtypes.

Interactions between CBFβ-SMMHC and the other CBFα subunits, RUNX2 and RUNX3, are tempting explanations for the differences between *RUNX1* mutated and inv(16) AML. Both RUNX2 and RUNX3 are expressed in adult hematopoietic stem and progenitor cells, and are predicted to heterodimerize with CBFβ-SMMHC [49]. Studies in mice show that decreased RUNX2 activity slows CBFβ-SMMHC induced leukemia, while increased RUNX2 expression accelerates it [79]. These findings imply that repression of RUNX2 by CBFβ-SMMHC is likely not the cause of the unique inv(16) AML phenotype. However, it is possible that the fusion protein alters RUNX2 activity in a way that contributes to the leukemogenesis.

How RUNX3 may contribute to the differences between the two leukemia subtypes is less well understood. *RUNX3* is frequently silenced by hypermethylation in inv(16) patient samples, and re-expression of RUNX3 decreases their proliferation *in vitro*. This implies that RUNX3 activity inhibits CBFβ-SMMHC induced leukemogenesis. [80]. How RUNX3 becomes hypermethylated and when it occurs in the leukemic process is not known.

### RUNX1 is necessary for CBFβ-SMMHC leukemogenesis

The observation that CBFβ-SMMHC has RUNX1 repression- independent activities raises the possibility that RUNX1 may be dispensable for CBFβ-SMMHC induced leukemogenesis. To test this possibility, mice expressing a conditional *Cbfb-MYH11* allele (*Cbfb^56M^*) under the control of the *Mx1-Cre Recombinase* (*Mx1-Cre^+^*) transgene were crossed with mice expressing a semi-dominant negative allele of *Runx1* in which the 3′ end of the gene is fused to the bacterial beta-galactosidase gene, *lacZ* (Runx1lz) [81]. The *Runx1^+/lz^* mice retain enough RUNX1 activity to bypass the embryonic lethality associated with nullizygous *Runx1^−/−^* mice, but have less RUNX1 activity than *Runx1^+/−^* mice. *Mx1-Cre*^+^; *Cbfb^+/56M^*; *Runx1^+/lz^* mice have a partial rescue of the differentiation and gene expression defects induced by the fusion gene [82]. In addition, *Mx1-Cre*^+^; *Cbfb^+/56M^*; *Runx1^+/lz^* mice show significantly delayed leukemia development as compared to *Mx1-Cre*^+^; *Cbfb^+/56M^*; *Runx1^+/+^* mice [82]. These findings demonstrate that Runx1 is required for efficient CBFβ-SMMHC induced leukemia.

The fact that the majority of the *Mx1-Cre*^+^; *Cbfb^+/56M^*; *Runx1^+/lz^* mice eventually develop leukemia does not necessarily mean that the fusion protein's RUNX1-independent activities are sufficient for leukemia development, if given enough time. These mice have one wildtype *Runx1* allele, so CBFβ-SMMHC:RUNX1 complexes can still form. Due to limited RUNX1, fewer of these complexes may assemble, and those that do form may have less transcriptional repression or activation activity. However, this residual RUNX1 activity may be enough to drive leukemia development.

Although the mechanism of RUNX1 activity is currently not understood, these results demonstrate a genetic requirement for *Runx1* for efficient CBFβ-SMMHC induced leukemia in mice. A similar requirement for RUNX1 has been demonstrated in the inv(16) patient derived cell line, ME-1. When short hairpin RNAs are used to knockdown RUNX1, ME-1 cells show decreased proliferation and increased apoptosis [83]. This confirms the importance of RUNX1 in CBFβ-SMMHC-expressing leukemia cells.

### A new model of CBFβ-SMMHC leukemogenesis

The finding that CBFβ-SMMHC has activities that don't involve RUNX1 repression, but that RUNX1 is required for leukemogensis has led to a new model of CBFβ-SMMHC activity. Rather than acting as a dominant repressor of RUNX1, it is possible that the fusion protein cooperates with RUNX1 to induce changes in gene expression [78, 82, 84]. In support of this model, immunohistochemistry of inv(16) patient samples demonstrate that CBFβ-SMMHC is localized to the nucleus [85]. In addition, chromatin immunoprecipitation (ChIP) experiments in ME-1 cells demonstrate that CBFβ-SMMHC and RUNX1 co-localize at genes whose expression is regulated by the fusion protein. When the fusion protein is knocked down in ME-1 cells, the majority of the genes bound by the fusion protein and RUNX1 show decreased expression, implying that the CBFβ-SMMHC:RUNX1 complex can act as a transcriptional activator [86]. Consistent with this observation, the majority of the genes deregulated in *Cbfb^+/MYH11^* embryos show increased expression as compared to wildtype littermates [78].

These observations do not exclude the possibility that CBFβ-SMMHC also acts as a transcriptional repressor of some target genes [78, 86]. CBFβ-SMMHC binds the transcriptional co-repressor Sin3a *via* its C-terminal repression domain (Figure 1) [87]. Therefore, it is possible that, in concert with Sin3a, CBFβ-SMMHC:RUNX1 directly represses expression of some target genes. In addition, some of these repression targets may be transcriptional repressors themselves. Silencing of transcriptional repressors by CBFβ-SMMHC may indirectly contribute to the overall increased gene expression caused by the fusion protein.

Using a model in which CBFβ-SMMHC directly regulates target gene expression, one would predict that the fusion protein requires heterodimerization with RUNX1 for its activity, and that with the loss of *RUNX1*, the fusion protein is no longer functional. However, it is also possible that the genetic requirement for *RUNX1* observed in CBFβ-SMMHC expressing cells is due to the activities of the wildtype CBFβ:RUNX1 heterodimer or to CBFβ-independent activities of RUNX1. Both the dominant negative *Runx1-lz* allele and shRNA knockdown of RUNX1 are predicted to affect all RUNX1 containing complexes [82, 83]. ChIP experiments in ME-1 cells identified genes associated with RUNX1 and wildtype CBFβ, as well as genes bound by RUNX1 alone. This implies that RUNX1 has transcriptional activities independent of the fusion protein in inv(16) leukemia cells [86].

It is likely that RUNX1 acts through multiple different protein complexes in inv(16) AML cells, and that the balance of these different activities influences the growth and survival of leukemia cells. This idea has been proposed to explain the unexpected finding that CBFβ-SMMHC fusions with reduced RUNX1 binding retain the ability to cause leukemia. Knockin mice have been generated that express a deletion mutant of CBFβ-SMMHC lacking the HABD (CBFβ-SMMHC _d179-221_) and with reduced RUNX1 binding affinity (Figure 1) [88]. Heterozygous mice expressing CBFβ-SMMHC _d179-221_ are viable, indicating that the loss of the HABD eliminates the embryonic lethality caused by dominant repression of RUNX1. Surprisingly, mice expressing CBFβ-SMMHC _d179-221_ rapidly develop leukemia, with significantly reduced latency as compared to mice with full length CBFβ-SMMHC [88]. In addition, a small number of inv(16) patients express fusion genes lacking the HABD, referred to as a type I fusion [89, 90]. The blast morphology and clinical course of patients with type I fusions are indistinguishable from patients with the more common fusions that include the HABD. Together, these findings indicate that high affinity binding of RUNX1 is not required for CBFβ-SMMHC induced leukemia [89, 90].

Currently the mechanism of accelerated leukemia development caused by the CBFβ-SMMHC _d179-221_ mutant is not known. One possible explanation is that the fusion protein transcriptionally regulates target genes that have both pro- and anti-leukemic effects, but that control of these genes requires different levels of CBFβ-SMMHC activity. The CBFβ-SMMHC _d179-221_ deletion mutant may retain enough activation and/or repression function to regulate genes associated with a pro-leukemic effect, but not enough activity to regulate anti-leukemic target genes. This would result in accelerated leukemia development. A similar mechanism has been demonstrated in the case of the RUNX1-ETO (also known as AML1-ETO or RUNX1-RUNX1T1) fusion protein, which is the product of the t(8;21) translocation. A splice variant of this fusion gene, RUNX1-ETO9a, lacks c-terminal co-repressor binding domains, and has reduced transcriptional repression and cellular dysregulation activity as compared to full length RUNX1-ETO. However, RUNX1-ETO9a causes accelerated leukemia development in mice and its expression is correlated with worse survival in patients [67, 91].

A second possible explanation for the accelerated leukemia development by the CBFβ-SMMHC _d179-221_ mutant is that it alters the amount of RUNX1 available to non-fusion protein complexes. Deletion of CBFβ-SMMHC's HABD may allow more RUNX1 to bind wildtype CBFβ or to participate in complexes with other transcription factors. This may result in increased expression of pro-survival genes, and an expanded pool of pre-leukemic cells capable of acquiring cooperating mutations [82, 83, 88].

The idea that the balance of RUNX1 activities in various complexes impacts CBFβ-SMMHC activity may explain the observation that loss of wildtype CBFβ causes accelerated leukemogenesis. Mice with one inducible *Cbfb-MYH11* knockin allele (*Cbfb*^56M^) were crossed with mice heterozygous for a null *Cbfb* allele (*Cbfb*^−^). The resulting *Cbfb^56M/−^* mice developed leukemia significantly faster than mice expressing the fusion protein in the presence of one wildtype *Cbfb* allele (*Cbfb^56M/+^*), indicating that wildtype CBFβ has activities that counter balance those of the fusion protein [21, 92]. Loss of wildtype CBFβ is expected to allow more RUNX1 to act in complexes with the fusion protein or with other transcription factors, and perhaps the activity of these other complexes accelerates the development of leukemia.

## TARGETING THE CBFβ-SMMHC:RUNX1 INTERACTION

Due to the importance of RUNX1 for CBFβ-SMMHC activity, efforts to develop inhibitors of the fusion protein have focused on disrupting the CBFβ-SMMHC:RUNX1 interaction. To date, two different small molecule inhibitors of the fusion protein have been identified, both of which are able to kill CBFβ-SMMHC expressing leukemia cells [8, 9]. Both current CBFβ-SMMHC inhibitors are unlikely to be used in patients due to poor pharmacokinetics and high effective concentrations. Nonetheless, these studies provide important proof-of-principal support for targeting the CBFβ-SMMHC:RUNX1 interaction for the treatment of inv(16) patients.

**Figure 2:**
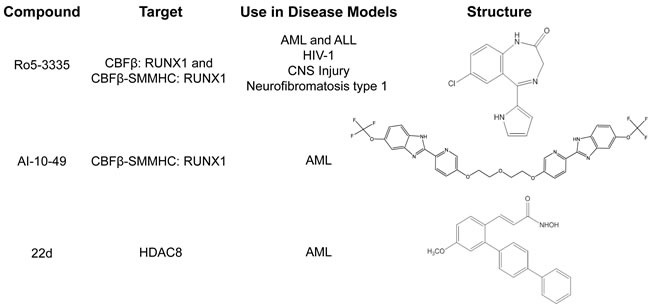
Small molecules targeting CBFβ-SMMHC Table listing current small molecule inhibitors targeting CBFβ-SMMHC, and other disease models each drug has been tested in. AML: Acute Myeloid Leukemia. ALL: Acute Lymphoid Leukemia. CNS: Central Nervous System.

**Figure 3:**
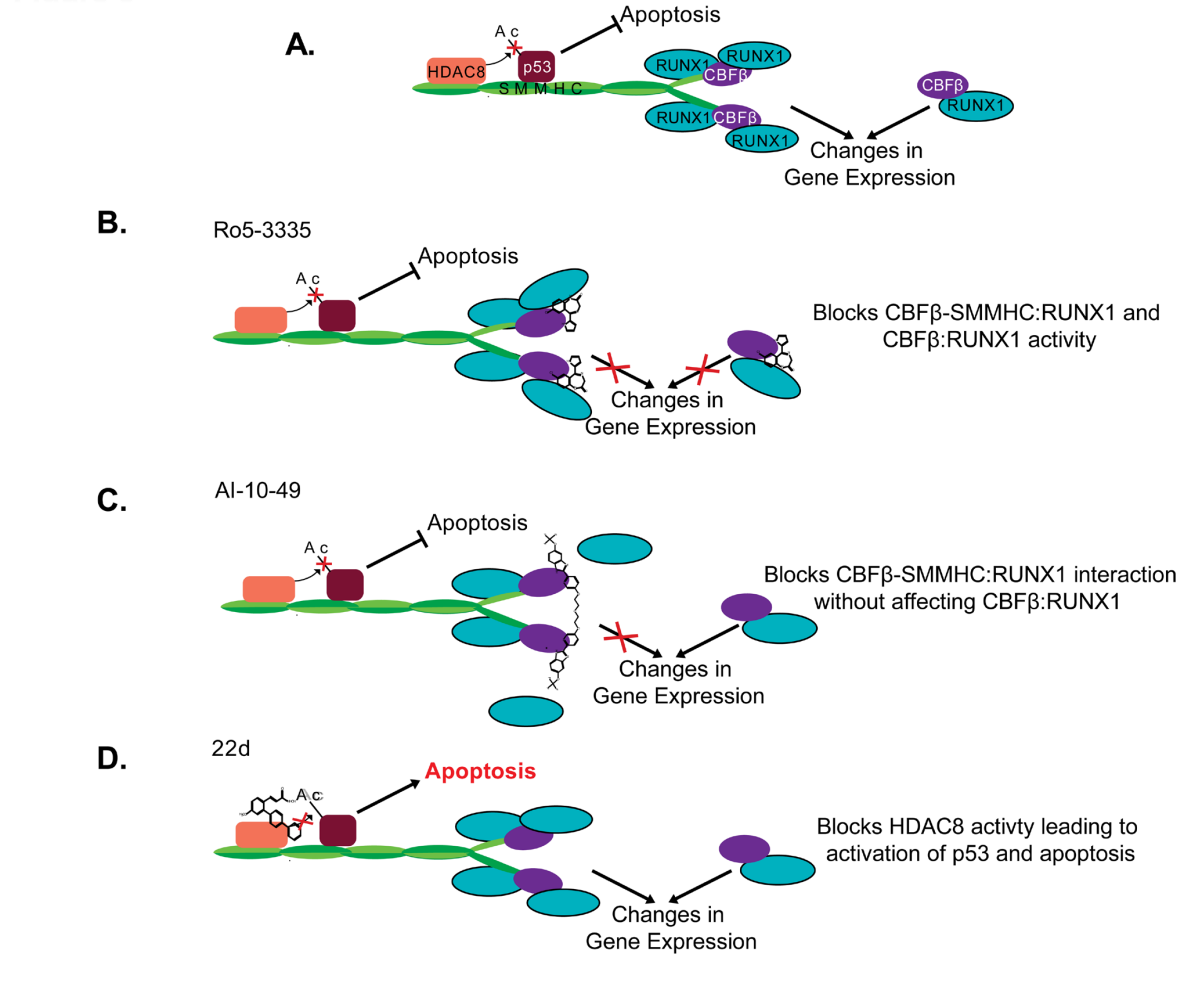
Proposed mechanism of CBFβ-SMMHC inhibitors Schematic representation of proposed CBFβ-SMMHC activities **A.** in the absence of inhibitors, or in the presence of **B.** Ro5-3335, **C.** AI-10-49, or **D.** 22d. Proteins are represented by the colored shapes labeled in (A). Ac: Acetylation group.

Ro5-3335 is a benzodiazepine that was identified in a screen for inhibitors of the interaction between wildtype CBFβ and the runt homology domain (RHD) of RUNX1 (Figures 2 and 3) [8]. Ro5-3335 binds both RUNX1 and CBFβ, and inhibits RUNX1 activity in a cell based promoter assay and in zebrafish. Treatment with Ro5-3335 reduces the viability of ME-1 cells. In leukemic mice, Ro5-3335 reduces disease burden and increases survival [8]. Because Ro5-3335 binds both RUNX1 and CBFβ, it is predicted to inhibit RUNX1 complexes with either the fusion protein or wildtype CBFβ (Figure 3). Extended treatment of wildtype mice with Ro5-3335 causes minor changes in platelet and red blood cell counts, and differences in white blood cell differentials. However, no obvious illness was observed, indicating that Ro5-3335 is relatively well tolerated in mice [8].

Currently, how Ro5-3335 inhibits RUNX1 activity is not known. *In vitro* assays with purified proteins show that Ro5-3335 blocks the interaction between CBFβ and the RHD. However, experiments with cellular extracts indicate that the drug does not prevent dimerization between full length RUNX1 and CBFβ, or binding to DNA. It is possible that Ro5-3335 causes a confirmation change in either RUNX1 or CBFβ which doesn't block heterodimerization, but alters the complex's DNA binding specificity or it's ability to activate and/or repress transcription [8].

Because Ro5-3335 inhibits RUNX1 and not just the CBFβ-SMMHC fusion protein, it may also be useful for the treatment of other leukemia subtypes that require RUNX1 activity (Figure 2). In fact, treatment with Ro5-3335 reduced viability in cells expressing other RUNX1 fusion proteins (RUNX1-ETO, and ETV6-RUNX1, also known as TEL-AML1), or the fusion protein MLL-AF9, which was recently shown to require RUNX1 for its leukemogenic activity [8, 93]. In addition, there are potential applications for Ro5-3335 in other diseases involving RUNX1, including Human Immunodeficiency Virus (HIV) infection, injuries involving the central nervous system, and Neurofibromatosis, type 1 [94-98].

A second small molecular drug targeting the CBFβ-SMMHC: RUNX1 interaction is called AI-10-49. In contrast to Ro5-3335, AI-10-49 specifically inhibits only the fusion protein (Figures 2 and 3) [9]. The parent compound for this inhibitor was identified in a screen for inhibitors of the CBFβ:RUNX1 interaction, and then subsequently shown to bind CBFβ and block its interaction with RUNX1. In order to generate an inhibitor that would preferentially interact with the fusion protein, a linker was used to generate a bivalent molecule. This strategy takes advantage of the observation that CBFβ-SMMHC exists as a multimer, while wildtype CBFβ is monomeric [71, 73, 99-101]. The bivalent inhibitor, AI-10-49, has a higher binding affinity for CBFβ-SMMHC than wildtype CBFβ. Treatment with AI-10-49 induces cell death in ME-1 cells, but in contrast to Ro5-3335, did not affect the growth of Kasumi-1 cells which express the RUNX1-ETO fusion protein. This indicates that AI-10-49 specifically inhibits the CBFβ-SMMHC:RUNX1 complex, and not the wildtype CBFβ:RUNX1 complex. Similarly, AI-10-49 did not significantly affect the survival or colony forming ability in culture of normal human hematopoietic cells *in vitro*. Importantly, treatment with AI-10-49 increased survival in mice with CBFβ-SMMHC induced leukemia, and decreased viability of primary inv(16) AML patient samples [9]. These results demonstrate that the bivalent inhibitor approach has potential for the treatment of inv(16) AML.

## CBFβ-SMMHC's C-TERMINUS

Initially, examination of CBFβ-SMMHC's activity focused on its interaction with RUNX1. More recently, domains in the C-terminus of the fusion protein have been shown to be relevant for leukemogenesis.

The C-terminus of CBFβ-SMMHC is composed of a series of α-helical repeats [71]. Work in cell lines has demonstrated that within this region, there is a multimerization or assembly competence domain (ACD) that allows for the self-dimerization of the fusion protein, and a repression domain that interacts with transcriptional co-repressors such as Sin3a and HDAC8 (Figure 1) [72, 73, 87, 100-102].

Studies in mice show the importance of CBFβ-SMMHC's C-terminus. Knockin mice expressing a CBFβ-SMMHC deletion mutant lacking the final 95 amino acids (CBFβ-SMMHCΔC95) show an almost complete rescue of the full length fusion protein's activity (Figure 1). In contrast to knockin mice expressing full length CBFβ-SMMHC, mice heterozygous for the CBFβ-SMMHCΔC95 allele (*Cbfb^+/ΔC95^*) are viable, with no obvious defects in primitive or definitive hematopoiesis [103]. Importantly, *Cbfb^+/ΔC95^* mice do not develop leukemia, even after treatment with the mutagen N-Ethyl-N-Nitrosourea (ENU). A small percentage of aged *Cbfb^+/ΔC95^* mice develop a non-transplantable myeloprolifeative disease (MPD). In addition, homozygous *Cbfb^ΔC95/ ΔC95^* embryos have similar differentiation defects during primitive hematopoiesis as described in *Cbfb^+/MYH11^* embryos [103]. As neither the MPD nor the primitive hematopoietic defect have been observed in mice with the null allele of *Cbfb*, these results indicate that the CBFβ-SMMHCΔC95 mutant retains some of the fusion protein's activity, but that the C-terminus is required for leukemogenesis. In more recent work, knockin mice expressing a CBFβ-SMMHC point mutant that specifically disrupts multimerization (*Cbfb^mDE^*) were generated. *Cbfb^+/mDE^* recapitulate many of the features of the *Cbfb^+/ΔC95^* including the failure to develop leukemia [101, 104]. This finding indicates that multimerization is required for CBFβ-SMMHC induced leukemia.

Another important CBFβ-SMMHC activity lost with the deletion of the terminal 95 amino acids is the interaction with the histone deacetylase, HDAC8 (Figure 2) [10, 87]. HDAC8 is an enzyme that catalyzes the removal of acetyl groups from lysine residues [105, 106]. Recent works demonstrates that the interaction between CBFβ-SMMHC and *HDAC8* leads to the deacetylation and subsequent inactivation of the transcription factor p53, which also binds CBFβ-SMMHC (Figure 3) [10]. Genetic experiments show that Hdac8 is required for CBFβ-SMMHC activity. Mice expressing the fusion protein but lacking HDAC8 show significantly delayed leukemia development. Importantly, treatment with the HDAC8 inhibitor 22d, an ortho-Aryl-N-hydroxycinnamide, selectively induced apoptosis in mouse and human CBFβ-SMMHC expressing leukemia cells. 22d had only minimal effect on the survival of non-inv(16) leukemia cells or CD34+ cells from healthy individuals [10, 107]. The precise mechanism of action for 22d is not known, but molecular modeling suggests that the drug interacts with a hydrophobic pocket at HDAC8′s active site, preventing the enzyme's interaction with substrates such as p53. In inv(16) leukemia cells, treatment with 22d prevents de-acetylation and subsequent inactivation of p53, allowing the transcription factor to remain in its active state and capable of inducing apoptosis [10, 107]. These results indicate that inhibition of HDAC8 is a potential strategy for the treatment of inv(16) AML.

## CONCLUSIONS

Although expression of CBFβ-SMMHC is known to be causative in inv(16) AML, the mechanism of its activity has been unclear. Recent advances demonstrate that the fusion protein's activity depends on at least two different interacting partners, RUNX1 and HDAC8 [10, 82, 83]. Importantly, pharmacological inhibition of either factor is effective at specifically targeting CBFβ-SMMHC expressing leukemia cells, with little effect on normal hematopoietic cells [8-10]. In addition, recent studies have pointed to additional targets for drug development. The importance of CBFβ-SMMHC multimerization and its inhibition of p53 are only beginning to be appreciated. Early studies indicate that both activities are critical for inv(16) AML and are intriguing avenues for future drug development [10, 104]. While much work remains, these recent advances indicate a promising future for the treatment of patients with inv(16) AML.
